# Klippel-Trenaunay Syndrome and Pregnancy

**DOI:** 10.1155/2010/706850

**Published:** 2010-12-19

**Authors:** Tuğba Güngor Gündoğan, Y. Jacquemyn

**Affiliations:** Department of Obstetrics and Gynaecology, Antwerp University Hospital UZA, Wilrijkstraat 10, 2650 Edegem, Belgium

## Abstract

Klippel-Trenaunay syndrome is a rare congenital vascular disorder, and only few cases have been described in pregnancy. We describe two cases, in one patient without complications, the other patient developed postpartum deep venous thrombosis.

## 1. Introduction

Klippel-Trenaunay syndrome is a congenital vascular disorder characterized by cutaneous hemangiomas, venous varicosities, and limb hypertrophy. Both obstetric and anesthetic management in these cases can be rather complicated. We report on two cases.

**Case 1**

A 33-year Caucasean woman had the diagnosis of Klippel-Trenaunay syndrome at age 5. As a child, some of the hemangiomata on the lower limb had been irradiated with radioactive cobalt. No further details on her childhood therapy could be found. Her body weight was 63 kg. She had never suffered any thrombosis.

During pregnancy, low-molecular-weight heparine nadroparin was started from 12 weeks on (nadroparin single daily subcutaneous dose 0.3 mL) (i.e., 2850 anti Xa, injection of Fraxiparine, Glaxo Smith Kline, Genval, Belgium), because the patient complained of progressive painful swelling and pain in the right leg; Doppler examination demonstrated no thrombosis.

At 36 weeks of gestational age, magnetic resonance image (MRI) of the lumbosacral region was performed which demonstrated some dilation of the anterior epidural venous plexus at the level lumbal 4-lumbal 5, but no arguments for an arteriovenous or venous malformation in the spinal canal.

At 39 weeks, she went into spontaneous labour and had a spontaneous delivery of a baby girl, weighting 2770 gram, Apgar 8 and 9 after 1 and 5 minutes. She received 10 IU of oxytocine as prevention for postpartum hemorrhage, this is a standard procedure for all patients in our center. The subcutaneous low-molecular-weight heparine was continued for six weeks after delivery. There were no problems in the postpartum period.

**Case 2**

A 36-year-old Caucasean woman presented for a first pregnancy. Diagnosis of Klippel-Trenaunay syndrome was made shortly after birth. She suffered from hypertrophy of the right leg 4 cm longer than left leg, atypically localized varices with an absent deep venous system at the right leg, and port-wine stains in multiple dermatomes on the right leg, abdomen, thorax, neck, face, and arms for which she had received cutaneous lasertherapy. There was no history of thrombosis. During pregnancy, no low-weight heparine nor low-dose acetylsalicylic acid was given. The patient did not demonstrate any change in her lower limbs during pregnancy. At 36 weeks, MRI of the lumbosacral spine proved to be impossible because the patient demonstrated severe claustrophobia.

At 40 weeks, she went in spontaneous labour and a boy, weighing 3690 gram, Apgar 8 and 9 after 1 and 5 minutes was spontaneously born. Immediately after delivery, low-molecular-weight heparine was started (Fraxiparine 0.3 mL, Glaxo Smith Kline, Genval, Belgium). She left the hospital on the fourth postpartum day and continued the use of nadroparin. On the 13th day, she developed acute phlegmasia alba dolens with complete thrombosis of the superficial veins of the right leg. Treatment consisted of compressive stockings and intermittent pneumatic compression together with a therapeutic dose of low-molecular-weigh heparine (Fraxodine 0.8 mL, Glaxo Smith Kline, Genval, Belgium, nadroparine 15200IE anti Xa once daily subcutaneously). On the same day under local anesthesia, a trombectomy was performed through an inguinal incision to remove the largest trombi on the level of the lateral and anterior side of the right upper leg. In one week time, there was spectacular diminishment of the swelling of the right upper leg which went from 10 cm difference with the left upper leg to 2 cm in circumference. The low-molecular-weight heparin and elastic compressive stockings were continued for six weeks after the event.

## 2. Discussion

Klippel-Trenaunay syndrome was first described in 1900 by two French physicians, Maurice Klippel and Paul Trenaunay. They called the disease naevus vasculosus osteohypertrophicus. The diagnostic criteria are capillary malformations, venous malformations and/or varicosities, and hypertrophy of the bone or soft tissues in one or more extremity [1].

Vascular disease can also be present in the internal organs such as the intestines or the uterus.

In 1907, Frederick Parkes Weber demonstrated that in this disease there was presence of arteriovenous fistula, since the syndrome has also been described as a Klippel-Trenaunay-Weber syndrome or angioosteohypertrophy syndrome [2]. Today, Klippel-Trenaynay syndrome is defined by the presence of a combined vascular malformation of the capillaries, veins, and lymphatics with congenital venous abnormalities and limb hypertrophy and is distinguished from Parkes-Weber syndrome in which an arteriovenous malformation with a cutaneous capillary malformation and skeletal or soft tissue hypertrophy occurs [3]. Both our patients demonstrated Klippel-Trenaunay syndrome.

The etiology of the syndrome is unknown. Several theories have been proposed including abnormalities of the sympathic nerve system resulting in dilatation of the arteriovenous anastomosis or obstruction of the deep veins and persistence of fetal microscopic small arterioveneous anastomosis [2].

Klippel-Trenaunay syndrome has a sporadic occurrence worldwide, we were unable to find any data linking the syndrome to specific ethnic groups. In about 1% of cases of Klippel-Trenaunay syndrome, a genetic pattern has been described, but the gene has not yet been identified [4–6]. It has been associated with an increased parental age and number of previous pregnancies [7]. The port-wine stains in Klippel-Trenaunay syndrome are mainly on the legs, less frequently on the arms of the trunk, and extremely rarely on the head or neck. They never exceed the midline. The varicosities on the legs result in a “Klippel-Trenaunay” vein. These varicosities increase in size during pregnancy. Hypertrophy of the soft tissues and bones leads to differences in leg length. Other described abnormalities include deep vein thrombosis, hypospadias, and lymph obstruction. At least two of three main symptoms (port-wine stains, varicosity, and hypertrophy of soft tissues and bones) must be present for the diagnosis Klippel-Trenaunay syndrome to be accepted.

Klippel-Trenaunay syndrome in a pregnant women is extremely rare, and only 19–20 cases have been published in the English language literature (summarized by Rebarber et al. [8] and 4 cases published later [9–12]). The normal physiologic changes of pregnancy, such as increased venous pressure, leg edema, venous stasis, and cardiac output, exacerbate the problems of this syndrome, resulting in an increased risk of thromboembolism and haemorrhage. Massive bleeding into the rectum, vagina, or vulva and vulvovaginal varicosities obstruction in the vaginal introitus have been described. An increased risk for intrauterine growth restriction has also been reported. As the disease can be hereditary in rare cases, the fetus should be checked for or the prenatal diagnosis of limb hypertrophy or multiloculated cystic lesions at the level of the thorax abdominal wall or limbs, what can be easily performed by fetal colour Doppler flow, which at the same moment can display eventual oaternal uterine angiomata, possibly complicating caesarean section. Different reports on prenatal diagnosis of Klippel-Trenaunay syndrome in fetuses have been published [13]. 

It has been suggested that these patients should be monitored for coagulopathies and screened for hereditary and acquired thrombophilic disorders. There is an ungoing discussion on the use of anticoagulant therapy including low-dose aspirine or low-molecular-weigh heparine during pregnancy. Due to increased swelling of the lower limbs, anticoagulant therapy was started in the first patient, in the second case, the anticoagulation was started only after delivery as the patient was considered high risk for postpartum thrombosis due to the varicosities. Despite the prophylactic low-molecular-weight heparin, she developed thrombosis; we do not know whether antepartum anticoagulant therapy could have prevented this complication. Coagulopathy is the most reported complication during pregnancy in women with Klippel-Trenaunay syndrome, including deep venous thrombosis and other tromboembolic problems both during and after delivery. Probably the risk is 10 times higher than in the normal population. There are no prospective trials on the use of anticoagulants and during pregnancy in Klippel-Trenaunay syndrome, so this should be discussed on an individual basis. If the patient had a tromboembolic accident in the past, therapeutic coagulation is indicated [14]. Antithrombotic stockings are generally advised. Kasabach-Merrit syndrome, which is a diffuse intravascular coagulation associated with major vascular malformations, has been reported [15].

 The presence of neuraxial vascular anomalies can complicate locoregional anaesthesia. It is advised to perform magnetic resonance imagining to detect angiodysplastic vascular structures next to the spinal cord (posterior cutaneous hemangioma, epidural and subdural vascular malformation) to avoid in these cases a traumatic puncture of these vessels. 

A multidisciplinary approach with the gynecologist, anesthesiologist, and vascular surgeon or haematologist forms the mainstay of the management of these patients.

There is no indication for cesarean section, except for the usual obstetric indication, cesarean section can be complicated by abdominal wall or uterine varices and hemangiomata. In the postpartum period, prophylactic anticoagulation is generally advised. 
